# Unhealthy eating habits among adolescent waterpipe smokers in Jordan: The Irbid-TRY study

**DOI:** 10.18332/tid/89976

**Published:** 2018-05-09

**Authors:** Nihaya A. Al-sheyab, Mahmoud A. Alomari

**Affiliations:** 1 Maternal and Child Health Department, Faculty of Nursing, Jordan University of Science and Technology, Irbid, Jordan; 2 Division of Physical Therapy, Department of Rehabilitation Sciences, Jordan University of Science and Technology, Irbid, Jordan

**Keywords:** cigarettes, waterpipe, smoking, adolescents, eating habits

## Abstract

**INTRODUCTION:**

The relationship between waterpipe smoking and eating habits among adolescents has not been investigated, thus the aim of the current study was to compare eating habits among adolescent cigarette-only, waterpipe-only, dual smokers versus non-smokers. We hypothesize that adolescent smokers practice unhealthy eating habits with worse habits among waterpipe smokers and dual smokers.

**METHODS:**

Using a descriptive, cross-sectional design, self-reporting patterns of waterpipe, cigarette smoking and eating habits were collected from a random representative sample of 1720 boys and girls in grades 7–10 in Northern Jordan.

**RESULTS:**

A total of 6.9%, 23.2% and 26.2% reported cigarette-only, waterpipe-only, and dual smoking, respectively. After controlling for gender, family income and education level of the mother and father, subsequent *post hoc* ANCOVA analyses revealed the following: mean (±SD) weekly breakfast consumption was greater in non-smokers (3.9±2.4) than waterpipe-only smokers (3.5±2.5, p=0.040) and dual smokers (3.2±2.5, p<0.000). Mean soda consumption frequency was lower in non-smokers (3.6±2.3) versus waterpipe-only (4.2±2.3, p=0.004) and dual smokers (2.7±2.2, p<0.000). Mean vegetable consumption was less in waterpipe-only (4.5±2.0, p=0.026) and dual smoking (4.4±2.0, p=0.013) smokers versus non-smokers (4.8±1.8). Mean consumption of energy drinks was the highest among dual smokers (1.2±1.9) versus non-smokers (0.5±1.4, p<0.000), cigarette-only (0.6±1.4, p≤0.01), and waterpipe-only (0.6±1.4, p<0.000) smokers.

**CONCLUSIONS:**

Overall, both waterpipe and dual smoking are associated with several unhealthy eating habits in adolescents. Given the growing epidemic of tobacco smoking in the Eastern Mediterranean Region, especially adolescent waterpipe smoking, and its association with unhealthy eating habits, public health actions and enforcing policies to decrease uptake of waterpipe smoking as well as improving food choices in schools among youth are urgently needed.

## INTRODUCTION

Adolescent tobacco consumption continues to be a public health problem^1^. In Jordan, for example, tobacco smoking among boys is one of the highest in the world^2^, while waterpipe and dual smoking (combined use of cigarettes and waterpipe) have exceeded that of cigarettes^3^. Specifically, the prevalence of waterpipe smoking among high school children in northern Jordan was 50%, which is higher than that of cigarette smoking^3^.

In 2014, waterpipe-only smoking was 22.9%, which was higher than cigarettes-only smoking (9.9%)^3^. A large recent cross-sectional study with 2407 Jordanian adolescents found that 57.6% reported smoking tobacco. Of those, the prevalence of dual smoking was 30%, which was higher than waterpipe-only (21.1%) or cigarette-only smoking (6.7%)^4^, suggesting a shift toward waterpipe smoking among adolescents over the last few years in Jordan. Further analyses of the same population found that waterpipe smoking is associated with greater obesity, BMI and body weight among Jordanian adolescents^5^.

In parallel, eating habits and practices of Arab countries have become more Westernized^6^. Previous studies of Arab adolescent lifestyle practices, including Jordanians, revealed that unhealthy food practices have become more prevalent recently^7,8^. These practices include inadequate intake of fruit, vegetables and milk, a high calorie/fatty diet, sugar-sweetened drinks, fast food, sweets, skipping breakfast, as well as very low levels of physical activity^7-9^. Similarly, a region-wide study found that only few adolescents consumed fruits and vegetables as frequently as guidelines recommend^10^. Additionally, several barriers to adopting healthy eating and adequate physical activity behaviors by Arab adolescents, including Jordanians^8^, were identified.

Although there is some research on clustering of lifestyle risk factors, there is still gaps in research on the youth population. To our knowledge, no studies have examined the relationship of waterpipe smoking with eating habits among Jordanian adolescents. Given the marked increase of waterpipe smoking and poor eating habits among adolescents, and their potential adverse health effects when combined, it is vital to investigate the associations between waterpipe smoking and eating habits among adolescents. Therefore, the aim of the current study was to compare eating habits among adolescent cigarette-only, waterpipe-only and dual smokers, versus non-smokers. We hypothesize that adolescent smokers practice unhealthy eating habits with worse habits among waterpipe smokers and dual smokers.

## METHODS

### Design and recruitment

The current data were derived from the Irbid Tobacco Risk in Youth (Irbid-TRY) study; a prospective, ongoing, longitudinal study designed to assess various health-related risks/conditions of tobacco consumption in adolescents. The current study used a cross-sectional design to examine the relationship between waterpipe smoking and eating habits among adolescent students, enrolled in Irbid-TRY, versus other types of tobacco smoking.

A simple random selection technique was used to randomly select 8 public high schools from two major educational districts in northern Jordan; Irbid 1st and Arramtha, which are a representative sample of school populations in northern Jordan.

Schools were stratified by gender to ensure equal representation of both sexes as public high schools in Jordan are single-sex. A two-stage cluster sampling design, in which the school and the class were the unit of cluster, was conducted as schools are considered a good venue to recruit students for research purposes. In each of the participating schools, an average of 4 classes of each grade with an average of 30 students within each class were randomly selected using a simple random technique. Within each of the selected classes, all healthy adolescents in the 7th to10th grades were invited to participate. The final response rate of participating students was 89.4% and 73.6% for smoking and diet patterns, respectively.

### Smoking status

Tobacco use patterns were obtained by a validated self-reporting survey in Arabic, adapted from the Youth Risk Behavior Survey^11^. Ever use of waterpipe or cigarette was assessed by two questions: ‘Have you ever smoked a cigarette’ and ‘Have you ever smoked a waterpipe’, respectively, with response options of ‘yes’ or ‘no’. Additionally, the survey asked two similar questions about waterpipe and cigarette smoking patterns: ‘Have you smoked waterpipe or cigarette in the past 30 days?’. The response options were: a) never used, b) have used, but not in the past 30 days, c) 1–2 days, d) 3–5 days, e) 6–9 days, f) 10–19 days, g) 20–29 days, and h) all 30 days.

To assess current (past month) smoking status, a 5-level current smoking status indicator variable was created: 1) never smokers (had never used either cigarettes or waterpipe), 2) non-current (ever) tobacco user (use of cigarettes and/or waterpipe but not in the past 30 days), 3) current (past 30 day) use of cigarettes but not waterpipe (cigarette-only), 4) current (past 30 day) use of waterpipe but not cigarettes (waterpipe-only), and 5) current (past 30 day) use of both cigarettes and waterpipe (dual smoking).

To assess frequency of use, 4-level indicator variables were created. For cigarette use frequency, the levels were: never smokers (never smoked either cigarettes or waterpipe); smoked cigarettes but not in the past month; smoked cigarettes in the last month but less than weekly; and smoked cigarettes weekly during the past month. Similar 4-level indicator variables were created to indicate the frequency of waterpipe smoking and dual smoking frequency.

Adolescents who reported either waterpipe/cigarette smoking, but not any other type of tobacco consumption in the past month were considered current waterpipe/cigarette smokers. Current dual smoking was classified for those who reported smoking both waterpipe and cigarettes in the past month.

### Eating habits

Eating habits were assessed using a self-reported survey; the Students As LifeStyle Activists survey (SALSA)^12,13^. The SALSA survey, originally developed in English, was translated into Arabic and back translated again into English by a professional translator. The survey had 58 items to obtain information about a variety of adolescent health aspects. It is a self-administered classroom activity that usually takes the students about 15 minutes to complete. The survey was facilitated by two research assistants and a volunteer school teacher.

The items concerning eating habits are used in the current study. These items include frequency of weekly consumption of fruit and vegetables; daily intake of fruit juice, water, soda and soft drinks; weekly consumption of junk food and fast food; and weekly frequency of having breakfast. The SALSA survey has no scoring system. The item responses were either ‘yes’ or ‘no’ or 4 to 5 Likert scale items. The research team undertook internal consistency reliability and validity analyses to obtain preliminary reliability and validity data about the questionnaire in an Arabic-speaking population (data are not yet published).

### Statistical analyses

SPSS software for Windows (version 22.0; Chicago, IL) was used for all statistical analyses. Data are expressed as means ± SD (standard deviation) for continuous data, while percentages and frequency are used for categorical variables. The alpha was preset at a significance level of p<0.05.

ANCOVA was used to compare differences in smoking patterns between the 4 smoking groups (i.e. none versus cigarette-only and waterpipe-only smoking), after controlling for student gender, family income and education level of the mother and father. Subsequently, LSD *post hoc* comparisons were used to verify specific differences between groups. Thereafter, a series of standard linear regressions were used to determine the individual relationship of smoking status (i.e. none versus cigarette-only and waterpipe-only smoking) with eating behavior. Additional stepwise regression that included the significant predictors found in the standard linear regressions was used.

## RESULTS

### Sample characteristics

A total of 2691 adolescents participated in the study, however only 2407 (89.4%) and 1771 (73.6%) reported data about smoking and diet patterns, respectively. Table 1 shows mean age, weight, height, BMI and school grade. As presented in Table 2, about 57.8% reported either dual (30.0%), waterpipe only (21.1%) or cigarette only (6.7%) smoking. Additionally, Table 2 shows that about 36.0% of the sample reported having breakfast at least 4 days a week, whereas 10.5% reported having no breakfast at all. About half of the sample reported eating vegetables (53.2%), fruits (45.2%) and dairy products (50.9%), 4–7 days a week. One the other hand, about half of the sample reported eating fried potatoes (59.9%), cakes and cookies (44.2%), chocolate and candy (55.6%), and soda (44.7%), at least 4 times a week.

**Table 1:** Participant Demographic Characteristics (n=2691)

*Demographics*	*% or Mean±SD*
**Gender (% female)**	63.0
**Grade (%)**	
7	23.2
8	22.3
9	28.8
10	25.7
**Age (years)**	14.6±1.1
**Weight (kg)**	56.8±13.8
**Height (cm)**	160.7±8.9
**BMI (kg/m^2^)**	21.8±4.3

**Table 2:** Smoking status and dietary consumption among participants

*Smoking status (%)*				
**Ever use of tobacco (n=2407)**				
Never	34.7			
Cigarette only	6.9			
Waterpipe only	23.2			
Dual	26.2			
***Diet behavior (%)***				*p*
**Eating habits (n=1771)**	**0 days**	**1-3 days**	**4-7 days**	
Breakfast	10.5	53.5	36	0.001
Vegetables	2.6	44.2	53.2	0.001
Fruits	3.2	51.7	45.2	0.001
Dairy product	11.7	37.5	50.9	0.001
Fast food	13	72.1	14.9	0.001
Fried potato	2.4	37.7	59.9	0.001
Cakes and cookies	5.3	50.4	44.2	0.001
Chocolate and candy	3.1	41.3	55.6	0.001
Energy drinks	74.9	18.4	6.7	0.001
Soda	6.4	48.9	44.7	0.001

### Relationship of tobacco smoking with eating habits

#### Comparisons

Table 3 shows average weakly dietary consumption among different smoking status categories, including none, cigarette, waterpipe and dual smoking. About 1720 adolescent students provided both sufficient smoking and diet data. After controlling for student gender, family income and education level of the mother and father, the ANCOVA showed differences in weekly consumption of breakfast (p<0.001), soda (p<0.000), vegetables (p=0.036), fast food (p=0.002), chocolate and candy (p=0.036), and energy drinks (p<0.000). As presented in Table 3, The ANCOVA showed the main effect of smoking status on weekly consumption of breakfast (p<0.001), soda (p<0.000), vegetables (p=0.036), fast food (p=0.002), chocolate and candy (p=0.036) and energy drinks (p<0.0001). Also, subsequent *post hoc* comparisons revealed the following: mean weekly breakfast consumption was less in waterpipe-only (3.5±2.5, p=0.040) and dual smokers (3.2±2.5, p<0.000) than non-smokers (3.9±2.4). Mean vegetable consumption during the week was less in waterpipe-only (4.5±2.0, p=0.026) and dual (4.4±2.0, p=0.013) smokers versus non-smokers (4.8±1.9). Weekly fast food consumption was higher in dual smokers (2.3±1.7) versus non-smokers (1.9±1.5, p<0.001) and cigarette-only smokers (1.7±1.3, p=0.004). Mean chocolate and candy weekly consumption was higher in dual smokers (4.4±2.1, p=0.040) versus non-smokers (4.0±2.2). Mean consumption of energy drinks during the week was highest among dual smokers (1.2±1.9) versus non-smokers (0.5±1.4, p<0.000), cigarette-only (0.6±1.4, p≤0.01), and waterpipe-only (0.6±1.4, p<0.0001) smokers. Mean soda consumption frequency was higher in waterpipe-only (4.2±2.3, p=0.004) and dual smokers (2.7±2.2, p<0.000) versus non-smokers (3.6±2.3).

**Table 3:** Differences in average weakly dietary consumption according to adolescent tobacco status (n=1720)

*Eating habits*	*None (n=752)*	*Cigarette (n=119)*	*Waterpipe (n=400)*	*Dual (n=449)*	*p*
Breakfast	3.9±2.4	3.4±2.5	3.5±2.5†	3.2±2.5†	0.001
Vegetables	4.8±1.9	4.4±1.9	4.5±2.0†	4.4±2.0†	0.040
Fruits	4.5±2.0	4.0±1.9	4.2±2.0	4.2±2.0	0.100
Dairy product	4.2±2.4	4.3±2.3	3.9±2.4	3.9±2.5	0.100
Fast food	1.9±1.5	1.7±1.3	2.0±1.6	2.3±1.7†‡	0.002
Fried potato	4.3±2.3	4.3±1.9	4.4±2. 3	4.3±2.2	0.870
Cakes and cookies	4.0±2.3	3.8±2.4	3.6±2.3	3.9±2.3	0.249
Chocolate and candy	4.0±2.2	4.4±2.1	4.1±2.2	4.4±2.1†	0.040
Energy drinks	0.5±1.4	0.6±1.4	0.6±1.4	1.2±1.9†‡	0.000
Soda	3.6±2.3	4.4±2.1	4.2±2.3†	2.7±2.2†‡*	0.000

Data presented as mean±SD.

† versus none;

‡ versus cigarette;

* versus waterpipe

However, the ANCOVA showed no differences in weekly consumption of fruits (p=0.100), dairy products (p=0.100), fried potatoes (p=0.900), and cakes and cookies (p=0.300) among smokers versus non-smokers (Table 3).

#### Regression

A series of standard linear regression analyses showed that smoking status predicted 6.7% (p<0.000) of eating behavior. However, only consuming breakfast (p<0.000), soda (p<0.000), fast food (p<0.001) and energy drinks (p<0.000) were significant. A stepwise regression that included the 4 significant factors, showed that smoking status predicted 6.8% of fast food (B=0.06, β=0.075, 95%CI=0.02/0.1, p=0.003), 6.1% of breakfast (B=-0.56, β=-0.111, 95%CI=-0.08/-0.033, p<0.000), 4.9% of energy drink (B=0.085, β=0.102, 95%CI=0.044/0.126, p<0.000), and 3.4% soda consumptions (B=0.82, β=0.150; 95%CI=0.056/0.109, p<0.000).

## DISCUSSION

The present study examined eating habits among adolescent tobacco smokers, including waterpipe. Overall, the study found that unhealthy dietary habits are often practiced by waterpipe-only and dual smokers compared to cigarette-only smokers and non-smokers. Differences in eating habits were found in consumption of breakfast, soda and energy drinks, vegetables, candy and chocolate. These findings are important and suggest that adolescent waterpipe smoking is associated with unhealthy eating habits. Our findings show that, among participating students, dual smoking was reported the highest, followed by waterpipe-only smoking, then cigarette-only smoking. More so, participating adolescents reported relatively high consumption of unhealthy food and beverages, such as fried potatoes, candy and chocolate, soda, as well as skipping breakfast.

These findings are very important and are congruent with the literature reporting that adolescent cigarette smokers are more likely to adopt an unhealthy dietary intake^14^. The current study, however, is the first to examine the relationship between waterpipe and dual smoking with eating behavior in adolescents. Similar to cigarette smoking^14^, our findings indicate that smoking waterpipe either alone or combined with cigarettes seems to be associated with unhealthy eating behaviors.

This association between unhealthy eating habits and waterpipe smoking is very alarming, especially in view that waterpipe smoking is spreading rapidly among adolescents. Studies have shown that waterpipe smoking is increasing in many countries worldwide, including Western countries^15,16^. Similarly, in Jordan, waterpipe smoking surpasses cigarette and dual smoking, which is also high^3^. In parallel to the increase in waterpipe and dual smoking among adolescents, Arab people, including youth, have adopted unhealthy eating habits^6-8^. Combined, a large cross-sectional study found that waterpipe smoking is associated with greater obesity, BMI and body weight among Jordanian adolescents^5^.

Waterpipe smoking is associated with adverse health consequences^17,18^, including an increase in oxidative stress and inflammation^19^. All these harmful effects are more likely to worsen if combined with unhealthy eating habits^20^. In particular, the current findings might also support evidence that adolescents who smoke cigarettes are more likely to be obese, which continues into adulthood^21^. Other evidence on adults found that waterpipe smokers tend to have higher abdominal obesity compared to non-smokers^22^. Furthermore, tobacco smoking was found to be associated with more physical inactivity^23^, and this could partially explain the clustering of unhealthy behaviors and the risk of obesity among waterpipe smokers in the current study.

Engaging in tobacco smoking has been attributed to a dampened appetite for healthy food^24^ and little concern about weight gain^25^. One important reason that could explain the prevalence of an unhealthy diet among adolescent smokers, especially those who smoke waterpipe, is the perceived appetite suppression effect of nicotine^26^. This may lead to weight control, which could be a main reason for continuing smoking and failure into relapses. The appetite-suppression effect of nicotine along with desire to control weight could also explain the habit of skipping breakfast among adolescents in the current study. However, published literature reported that people who smoke do not eat less than those who do not smoke^27^. In fact, smoking and nicotine can increase the metabolic rate, although cannot largely explain the change in weight following the change in smoking status/pattern (i.e. stopped smoking)^28^. Similarly, a reduction in weight can only be noticed temporarily among people who newly initiated smoking or smokers who experience relapses. Nonetheless, policies on cigarette and waterpipe smoking prevention are not fully endorsed in Jordan, and also not in compliance with the FCTC requirements with regards to Article 11, and lag behind regionally and worldwide^29^. Although the law requires health warnings on all tobacco products, health warnings in Jordan have been authorized only for cigarette packs^29^.

Importantly, since waterpipe smoking has a strong social underpinning, community-based and peer-led interventions could be developed to renormalize this behavior in the region. Finally, the current study shows that adolescent smokers, particularly waterpipe and dual, are adapting unhealthy eating habits. Accordingly, smoking cessation programs should also consider eating behavior modifications. In fact, given that unhealthy behaviors tend to cluster, adolescent smokers should attend comprehensive behavior modification programs that target other unhealthy behaviors.

### Limitations

The current study has some limitations. Tobacco smoking and eating patterns were self-reported, which might be associated with false reporting. However, the Arabic version of the Youth Risk Behavior Survey is found valid and a reliable measure for smoking behavior among adolescents^11^. Future studies need to use biological markers to assess smoking status to confirm the relationship of waterpipe smoking and dual smoking with eating habits. The current study used a cross-sectional design, and thus cannot draw any causal inferences. Therefore, longitudinal research is needed to confirm the current findings. Moreover, the current study was conducted in northern Jordan, thus limiting the generalizability of the findings to other countries. However, the pattern of waterpipe and dual smoking tend to be similar among adolescents, especially in the Middle East and North Africa (MENA) region^30^.

## CONCLUSIONS

Unhealthy eating habits are practiced among adolescent smokers, especially those reporting waterpipe-only and dual smoking, compared to cigarette-only smoking and non-smoking. Public health action as well as developing and enforcing relevant policies for waterpipe smoking and healthy food choices are urgently needed to reduce waterpipe smoking and increase awareness about the combined negative effects of waterpipe smoking and unhealthy dietary choices, especially among youth. This is particularly important because waterpipe smoking has become an epidemic, with waterpipe exceeding cigarette smoking, and dual smoking also being high, especially among girls.
